# Insights into the Pathogenesis of Enteropathogenic *E. coli* Using an Improved Intestinal Enterocyte Model

**DOI:** 10.1371/journal.pone.0055284

**Published:** 2013-01-28

**Authors:** Paul Dean, Lorna Young, Sabine Quitard, Brendan Kenny

**Affiliations:** Institute of Cell and Molecular Biosciences, Medical School, University of Newcastle, Newcastle-Upon-Tyne, United Kingdom; University of Glasgow, United Kingdom

## Abstract

Enteropathogenic *E. coli* (EPEC) is a human pathogen that targets the small intestine, causing severe and often fatal diarrhoea in infants. A defining feature of EPEC disease is the loss (effacement) of absorptive microvilli (MV) from the surface of small intestinal enterocytes. Much of our understanding of EPEC pathogenesis is derived from studies using cell lines such as Caco-2 – the most extensively used small intestinal model. However, previous work has revealed fundamental differences between Caco-2 cells and *in vivo* differentiated enterocytes in relation to MV effacement. This, and the high heterogeneity and low transfection efficiency of the Caco-2 cell line prompted the isolation of several sub-clones (NCL-1–12) to identify a more tractable and improved *in vivo*-like cell model. Along with established Caco-2 clones (TC-7, BBE1), sub-clones were assessed for growth rate, apical surface morphology, epithelial barrier function and transfection efficiency. TC-7 cells provided the best all-round clone and exhibited highest levels of ectopic gene expression following cell polarisation. Novel alterations in EGFP-labelled mitochondria, that were not previously documented in non-polarised cell types, highlighted the potential of the TC-7 model for defining dynamic enterocyte-specific changes during infection. Crucially, the TC-7 cell line also mimicked *ex vivo* derived enterocytes with regard to MV effacement, enabling a better dissection of the process. Effacement activity caused by the EPEC protein Map in the Caco-2 but not *ex vivo* model, was linked to a defect in suppressing its Cdc42-dependent functionality. MV effacement activity of the EPEC protein EspF in the TC-7 model was dependent on its N-WASP binding motif, which is also shown to play an essential role in epithelial barrier dysfunction. Together, this study highlights the many advantages of using TC-7 cells as a small intestinal model to study host-pathogen interactions.

## Introduction

Cell lines have been instrumental to our understanding of cell biology and disease. While several intestinal cell lines are available, Caco-2 is by far the most commonly used model of the small intestine [1], [2] – employed in a broad range of fields such as pharmacology, nutrition, toxicology and host-pathogen studies [3], [4]. Caco-2 cells spontaneously differentiate in culture to express prominent features of enterocytes including a microvilli brush border, tight junctions, many enterocytic enzymes and transporters [1], [5], [6], [7]. Despite their popularity, one of the major limitations with the Caco-2 model is its heterogeneity, as the parent cell line is comprised of a mixed cell population. This has a significant impact on reproducibility of results as Caco-2 cells derived from different sources may be made up of a vastly different cell populations [1], [4], [8]. Extrinsic factors such as culture conditions, seeding densities and cell passage may also favour specific sub-populations of cells [1], [4], making the parental Caco-2 line less reliable, and making comparisons of data from different labs more difficult [8]. Furthermore, visualisation of the Caco-2 cell surface at the single cell level is complicated due to the mixed cell population. To alleviate these problems, Caco-2 clones have been isolated and characterised by several laboratories [9], [10], [11].

Enteropathogenic *E. coli* (EPEC), which causes severe watery diarrhoea, specifically targets the human small intestine [12], [13]. Like many other enteric bacterial pathogens, EPEC delivers over 20 virulence-related effector proteins directly into the host cell cytoplasm using a dedicated type three secretion system (TTSS). Although effectors are critical for EPEC disease, most of our knowledge on their function is derived from work using non-intestinal cell types such as HeLa – that are unable to differentiate into a polarised epithelium. EPEC belongs to a group of enteric pathogens that causes attaching and effacing (A/E) lesions on the surface of intestinal cells – a key feature of A/E pathogen disease [12], [14]. A/E lesions are caused by the effacement of host microvilli along with the formation of an actin-rich pedestal beneath the bacterium. Pedestal formation is dependent on the effector protein Tir which, upon delivery into the host cell, inserts into the host plasma membrane to act as a receptor for the bacterial outer membrane protein Intimin [15]. We previously showed that microvilli effacement is caused by the combined action of 3 effectors (Map, EspF and Tir) delivered into the host cell [16]. However, this work revealed a crucial difference between the effector-driven signalling in Caco-2 and *ex vivo* derived intestinal biopsy tissue as the EspF effector effaced microvilli in both tissue types whereas Map only effaced in the Caco-2 model [16].

In this study, we attempted to identify Caco-2 clones that exhibited *in vivo*-like features of the host-pathogen interaction while also providing greater experimental tractability. Clones were tested for transfectability, morphology, growth rate and epithelial barrier function and whether they supported *in vivo*-like signalling with regard to microvilli effacement. The high transfectability of some clones, even when well differentiated, enabled a real time analysis of target cell types during EPEC infection. This work uncovered novel effector functions within polarised cells relating to the host mitochondria, EPEC-mediated microvilli effacement and epithelial barrier dysfunction.

## Materials and Methods

### General procedures, cell lines, plasmids and bacterial strains

Bacterial strains and plasmids used in this study are given in Table 1. Bacteria were cultured at 37°C on Luria-Bertani (LB) agar plates taken from glycerol stocks. Prior to infections, bacteria were grown at 37°C as standing cultures overnight in LB broth with appropriate antibiotics (nalidixic acid, kanamycin or carbenicillin 50, 25 and 100 µg/ml final concentration respectively). Mammalian cell lines were Caco-2 (ATCC HTB-37), TC-7 [10] and BBE1 [11]. Cell clones NCL1-12 (this study) were isolated as described below. All cells were maintained in 75 cm^2^ flasks using standard tissue culture procedures as previously described [17]. Every 2 days cells were given fresh Dulbecco's minimal Eagle medium (DMEM; Invitrogen) supplemented with 2 mM L-glutamine (Sigma), 1% non-essential amino acids (Sigma), 1× penicillin/streptomycin (Sigma) and 10% (v/v) heat inactivated foetal calf serum (Gibco). Prior to confluence, cells were trypsinised for 20 min and the resulting suspension was seeded into a new 75 cm^2^ flask, onto a coverslip or membrane filters as previously described [17].

**Table 1:** Bacterial strains, plasmids and cell lines used in this study.

Enteropathogenic *E. coli* strain	Description	Source/Reference
Wild type EPEC	Prototypical strain E2348/69	[38]
Δ*map*	Lacking the *map* gene	[18]
Δ*espF*	Lacking the *espF* gene	[39]
Δquad	Lacking map, *espF*, *tir* and *eae* (encodes Intimin) genes	[40]
Δ*mapespF*	Lacking map and *espF* genes	[17]
ΔTTSS	Type three secretion mutant deletion for *espA*	[41]
**Plasmids**	**Description**	**Source/Reference**
pd2-CMV-EGFP	Mammalian EGFP expression	This study
pEGFP-MITO	Mitochondrial targeting sequence of cytochrome c fused to EGFP	[23]
p*espF*	pBR322 vector for bacterial expression of *espF*	[20]
p(L16E)*espF*	pBR322 vector for bacterial expression of *espF* missing the mitochondrial localisation sequence	[20]
p*espF*(D3)	the critical arginines of *espF* in the three SNX9 motifs exchanged for aspartic acid	[28]
p*espF* (A3)	*espF* expression with all three critical leucine in the N-WASP binding motif exchanged for alanine	This study
p*espF* (A2)	As above, with only 2 leucine exchanged – L104A and L197A	This study
p*espF* (A1)	As above, with only 1 leucine exchanged – L104A	This study
pTccp	Bacterial expression – *espF* homologue Tccp	[23]
pSK-*map*	pBluescript based bacterial expression vector for *map*	[18]
pSK-*map*ΔTRL	pSK-map with the C-terminal DTRL amino acids removed	This study
pSK-*map*ΔE78A	pSK-map with the E78A substitution	Thus study
		
**Cell lines**	**Description**	**Source/Reference**
Caco-2	Heterogeneous model for small intestinal enterocytes.	ATCC HTB-37
TC-7	Homogeneous Caco-2 clone	[10]
HeLa	Human cervical carcinoma cells	ATCC No. CCL-2
Clones NCL1-NCL12	Caco-2 clones	This study
BBE1	Caco-2 clone with dense brush border	[11]
**Primers**		
P1-E78A	GCAATGGTTCAAGCAAGCGCAGATTACTTTTCTATCC	
P-ΔTRL PS	CGGAATTCATGTTTAGTCCAACGGC	
P-ΔTRL NS	CGGTCGACCTAATCCTGCACATTGTC	
P-EspF NW NS	CGATAGTTCATAGGCAGCTGCTGCATGATCTTTTAGTGCCTGTGC	

### Immunoblotting to detect translocated effector proteins

Infected cells were washed with cold Phosphate Buffered Saline pH 7.4 (PBS) and lysed with 0.2% Saponin (w/v) to release the cytosolic fraction. Cells were sedimented (13,000 g×5 minutes) and the pellet was solubilised with 1% (w/v) Triton X-100 to release the membrane fraction followed by centrifugation (13,000 g×5 minutes). All lysis solutions contained a protease inhibitor cocktail (Sigma). The protein concentration of the cytoplasmic and membrane fractions were determined using Bradford reagent and 20 µg of protein was resolved by 12% SDS PAGE. Proteins were transferred to nitrocellulose, blocked in 5% milk powder/PBS and probed with antibodies against Tir, EspF and EspB (obtained commercially from Dr Roger James Department of Surgery, Leicester University). Western blot was carried out using an alkaline phosphatase-conjugated secondary rabbit antibody (Jackson Laboratories) and the signal was developed using the NBT/BCIP reagent (Pierce).

### Scanning electron and confocal microscopy

Polarised epithelial cells were infected with bacterial strains as previously described [16] using an MOI of 1∶30. Scanning electron microscopy was carried out as described previously [16]. Quantification of microvilli effacement was carried out using the freely available software package Image J by determining the percentage level of peripheral effacement in randomly selected fields from 3 separate experiments. Individual microcolonies were assessed for their ability to cause peripheral microvilli effacement; expressed as percentage of the total microcolony count. Indirect immunofluorescence was carried out using a Leica SP2 confocal microscope. Polarised cells grown on membrane filters (Corning) perforated with 0.4 µm pores were fixed for 15 min in 2.5% paraforamaldehyde/PBS and permeabilised in 0.2% Triton X-100. Cells were stained with DAPI (Invitrogen), phalloidin (Alexa 594-conjugated; Invitrogen) and immuno-stained using rabbit antibodies for occludin (Abcam) or ZO-1 (Abcam). These were detected with secondary an anti-rabbit antibodies conjugated to alexa-488 (Invitrogen).

### Clonal selection of Caco-2 cells

Selection of Caco-2 clones was carried out by limited dilution. Cells in exponential phase were trypsinised and serially diluted into 150 mm culture dishes (Corning) in culture medium. Single adherent cells were identified by microscopy and after several days of growth, colonies (3–6 mm diameter) were isolated using 10 mm cell cloning cylinders (Millipore). Colonies were trypsinised and sub-cultured, first in 24-well plates, then in tissue culture flasks. Clones were used after 3 passages and frozen for long term storage.

### Ectopic gene expression in polarised Caco-2 clones

Cell cultures were trypsinised as above and diluted in DMEM (without supplements) to a concentration of 2×10^6^ cells/mL in suspension. A lipofectamine 2000 (Invitrogen):plasmid DNA mix (made according to the manufacturer's instructions except the DNA concentration was three times the suggested level) was incubated with the cell suspension then rotated at 37°C for 30 minutes. Cells were immediately sedimented onto 13 mm sterile glass coverslips within a 24-well plate (Corning) by centrifugation at 500 g for 5 minutes. Following subsequent incubation for 6 h at 37°C, the medium was replaced with complete DMEM. Using this method, adherent cells were confluent by 24 h post-transfection. The following day, adherent cells were transfected once more using the same liposome:DNA concentrations as described above, according to the manufacturer's instructions. Transfected cells were then left to polarise for 10–15 days.

### Transepithelial electrical resistance (TER) and growth determination of Caco-2 clones

For TER studies, cells were grown on permeable polyester filters with a 0.4 µm pore size (Corning) for 12–15 days and infected with the indicated EPEC strains as described previously [17]. The electrical resistance was measured at 37°C over a 6 h post-infection period using an EVOM epithelial voltohmeter (World Precision Instruments). Growth rate of the clones was determined in a 24 well plate seeded with 1×10^5^ cells. When the fastest clone (clone NCL4) had reached 100% confluence, the confluence of the remaining clones was determined by microscopy over 8 fields of view at x400 magnification.

### Generation of bacterial and mammalian expression vectors

Point mutation of Map in its WxxxE motif involved substituting the critical glutamic acid at position 78 for an alanine residue (E78A). Mutagenesis was performed with the QuikChange Site-Directed Mutagenesis kit (Stratagene) using pSK-*map* [18] as a template with the primer P1-E78A (Table 1) according to the manufacturer's instructions. To remove the last 4 amino acids of Map, the *map* variant *map*ΔDTRL was constructed by amplifying the *map* gene using pSK-map as a template with primers P-ΔTRL PS and NS (Table 1) which re-introduced a stop codon. The amplified product was subcloned into pSK-bluescript using EcoRI and SalI restriction sites. Mutation of the N-WASP binding motif (NWBM) in EspF was performed by substituting the critical leucine residues (positions 104, 151 and 197) required for N-WASP binding and actin nucleation [19] for alanine in each NWBM. This was performed using the QuikChange Multi Site-Directed Mutagenesis Kit (Stratagene) with the primer P-EspF NW NS (Table 1) and using pBR322-*espF* [20] as a template. Leucine residues were substituted in single, double and triple combinations and a PvuII restriction site was introduced for screening purposes. The mammalian expression vector pd2CMV-EGFP was constructed by subcloning the Not1/EcoRI fragment of pd2EGFP [21] containing the EGFP gene into the Not1/EcoRI restriction sites of pEGFP-N1 (Clontech) – thus placing the EGFP under a constitutive CMV promoter. All new constructs were verified by sequencing.

### Statistical analyses

Unless stated otherwise, experiments were repeated independently at least 3 times. Data represents the mean ± SEM and where necessary, comparison of means was performed using a one-way ANOVA with a post-hoc Tukey test to indicate significant differences between data points; p values less than 0.05 taken as a significant difference.

## Results

### Isolation and characterisation of Caco-2 clones

The mechanism of EPEC-mediated microvilli effacement fundamentally differs between enterocytes differentiated *in vitro* (Caco-2 model) and *in vivo* [16]. We speculated that this discrepancy may relate to the heterogeneity of the Caco-2 model, thus subclones were isolated (NCL1-12; see Materials and Methods). These were assessed for surface morphology (by scanning electron microscopy), ability to polarize and form a transepithelial electrical resistance, growth rate and transfection efficiency. In addition, two well established Caco-2 clones, TC-7 [10] and BBE-1 [11], along with the parental cell line were included in our analyses.

**Figure 1:**
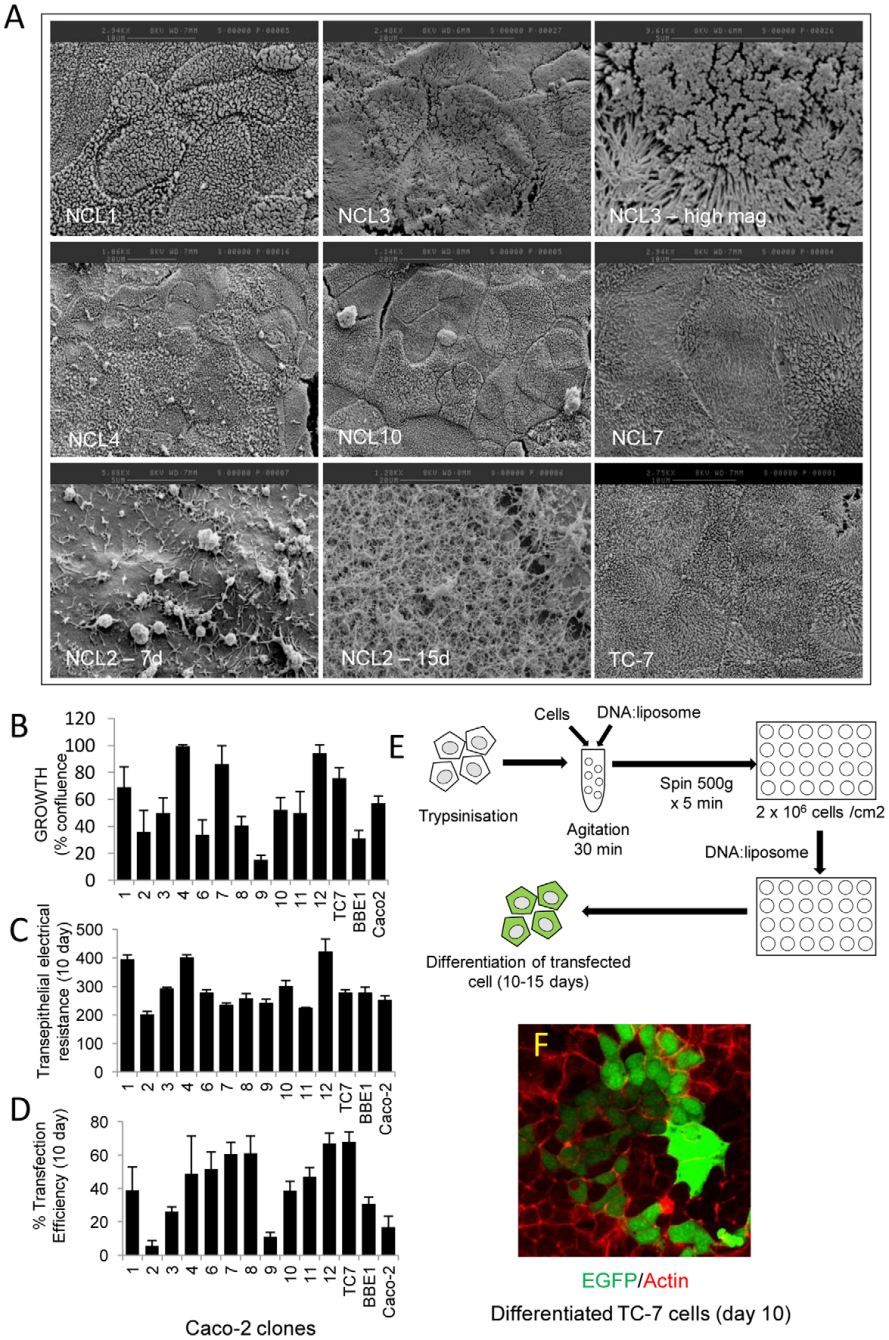
Characterisation of Caco-2 clones. Twelve clones (NCL1-12) were isolated by limited dilution and assessed by electron microscopy (A), growth rate (B), electrical resistance (C) and transfection efficiency (D). The high magnification of clone NCL3 shows the long microvilli (A) while clone NCL2 at 15 day post-confluence was covered in a glycocalyx-like material. A modified transfection protocol (E) was employed to achieve high levels of transfection of the clones. Transfection efficiency (D) was determined in polarised host cells (10 days) following expression of EGFP under a constitutive CMV promoter (F). All data points represent means ± SE, n = 3.

Visual assessment of the clones by scanning electron microscopy (SEM) revealed homogeneous populations (Figure 1A) although two clones (NCL8 & 9) exhibited variations of their apical surface linked to their stage of differentiation. Clone NCL5 deteriorated with increasing passage and was eventually lost so was not included in our analysis but all other clones remained stable following passage. Five clones exhibited a well packed brush border (TC-7, NCL3, NCL6, NCL10 & NCL12; Figure 1A), similar to that of *in vivo*-differentiated enterocytes [16], [22]. Notably, NCL3 had unusually long microvilli (Figure 1A). By contrast, NCL2 was completely devoid of microvilli, with a denuded surface covered by a filamentous mesh that became thicker during differentiation (Figure 1A) – presumably representing a glycocalyx-like material. The growth rate and TER values for all the clones was also measured, revealing considerable variation between them (Figure 1B and C), reinforcing the view that the Caco-2 population is highly heterogeneous.

### Transfection efficiency of Caco-2 clones upon polarisation

**Figure 2:**
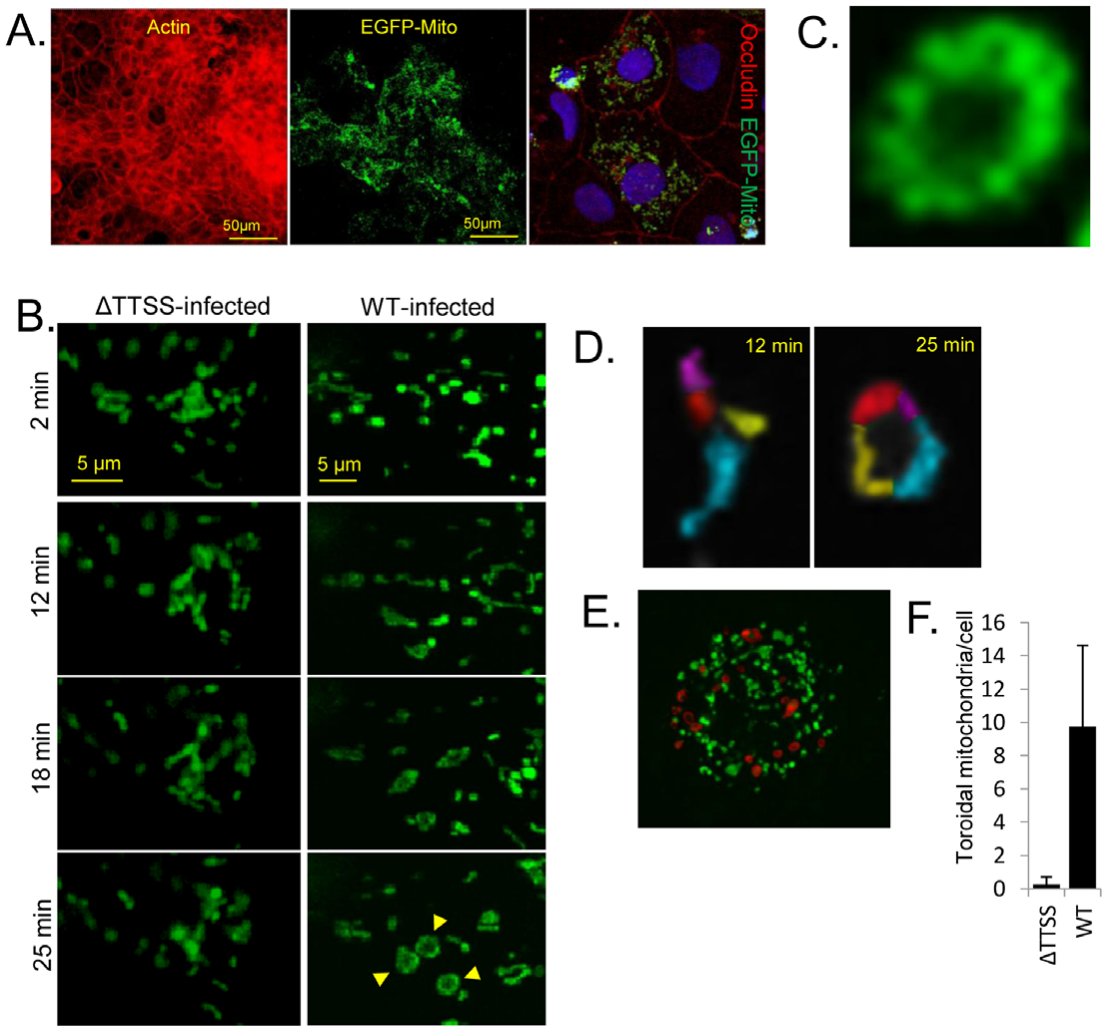
EPEC induces toroidal-shaped mitochondria in polarised TC-7 cells. Expression of a mitochondrial-targeted EGFP protein in polarised TC-7 cells shows a high level of transfection efficiency with occludin staining pattern confirming polarisation (A). Live cell imaging of EGFP-labelled mitochondria was performed in polarised TC-7 cells infected with a type three secretion system (ΔTTSS) defective mutant (*espA*) or wild type (WT) EPEC (B). Selected image captures are given with the infection time (left) (B). Captured images show the spatial organisation of mitochondria in cells infected with the two mutants. Arrows show the doughnut shaped (toroidal) mitochondria (B). Toroidal mitochondria were derived from a fusion of individual mitochondria (pseudocoloured; D). Late stage infected cells (60 min) containing many toroidal mitochondria (pseduocoloured red; E), quantified in (F). Toroidal-shaped mitochondria were defined as a continuous ring surrounding a central void. Data represents the mean ± SD, n = 3.

As with non-polarised cell types, a transfectable intestinal model is highly desirable for studying host-pathogen interactions, particularly if this model can polarise. Preliminary work using an EGFP-expression vector indicated that it was not possible to transfect pre-polarised (7–15 days) cells (not shown). Therefore, cells were transfected in suspension in their non-polarised state prior to seeding at high confluency (see Figure 1E). Transfected cells were left to differentiate for 10–15 days, revealing that a small sub-population of the parental Caco-2 cell line (<20%), retained the ability to express EGFP following differentiation, while the individual Caco-2 clones displayed much higher (and lower) levels of transfection (Figure 1D). The TC-7 cell line in particular gave the highest transfection efficiency with over ∼60% EGFP positive (Figure 1D–F). Immuno-staining TC-7 cells (Day 10 post confluence) for the tight junctional protein ZO-1 (data not shown) and occludin (see Figure 2A) supported the TER data that the cells were polarised. As TC-7 cells exhibited (i) the highest transfection efficiencies of all the clones tested (ii) relatively quick doubling time (iii) high TER and (iv) densely packed (*in vivo*-like) brush border and (iv) have been well characterised by others [1], [10], they were put forward for EPEC infection studies.

### Ectopic gene expression reveals dynamic alterations in polarised enterocyte mitochondria during EPEC infection

We recently reported that fixed-cell imaging of polarised cells which express fluorescently-tagged EPEC effectors or epithelial proteins can provide insights on both epithelial and pathogen biology [23], [24]. However, little is known about dynamic pathogen-induced changes in polarised cells during infection. Given that TC-7 cells were amenable to transfection, we expressed an EGFP fusion protein (EGFP-MITO) in the TC-7 model to label host mitochondria (Figure 2A) as these organelles are known to be targeted by EPEC during infection [25]. Transfection of TC-7 with EGFP-MITO resulted in a high level of ectopic gene expression after polarisation (Figure 2A), supporting the EGFP data (Figure 1F). Infection with an *espA* EPEC mutant, which is defective in a functional type three secretion system (ΔTTSS), revealed that mitochondria oscillated short distances with regular frequency (not shown) and maintained a relatively consistent shape (Figure 2B). Indeed, the overall spatial pattern of mitochondria in *espA*-infected cells exhibited little change whereas infection with wild type EPEC resulted in greater mitochondrial movement (Figure 2B). The most striking phenotype associated with wild type EPEC infection was the formation of large toroidal (doughnut) shaped structures (Figure 2B and C) – formed due to mitochondrial end-to-end fusion (Figure 2D). These structures were not evident in *espA*-infected TC-7 cells (Figure 2B and F), suggesting that they were induced by a delivered bacterial effector(s) protein. Toroidal-shaped mitochondrial structures increased progressively during infection and were present in high numbers in late-stage infected cells (pseudo-coloured red; Figure 2E and F). This data shows that real time imaging of polarised TC-7 cells can provide novel insights into the dynamic changes within target host cells during infection.

### An improved *in vivo*-like model for studying EPEC-mediated microvilli effacement

**Figure 3:**
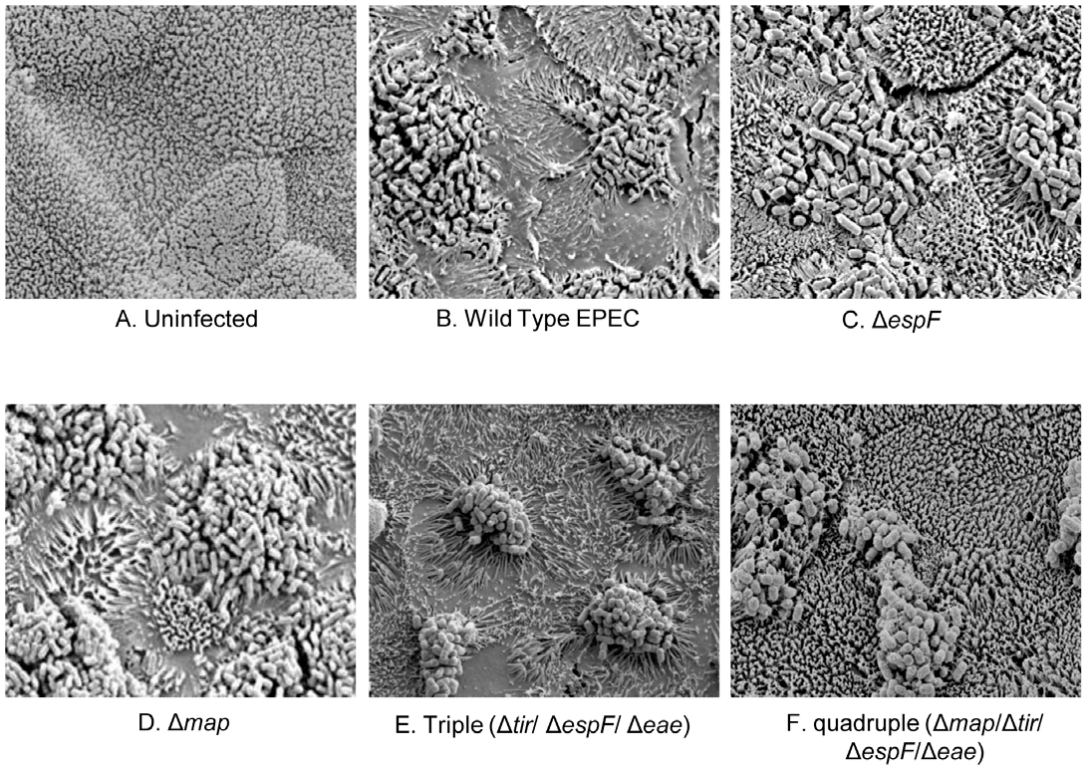
EPEC effacement on TC7 cells mimics *in vivo*-differentiated enterocytes. Scanning electron microscopy analysis of TC-7 cells reveals a highly homogeneous densely packed brush border (A) that is effaced by wild type EPEC (B) over a 120 minute infection period. No peripheral effacement activity was detected during infection with the Δ*espF* mutant (C) whereas the Δ*map* mutant behaved like the wild type strain (D). A triple mutant (Δ*espF*Δ*tir*Δ*eae* [latter encodes Intimin])hyper-effaced microvilli (E) with removal of Δ*map* from this strain abolishing effacement activity (F).

Previous reports show that the mechanism of EPEC effacement is different in Caco-2 and *in vivo* differentiated enterocytes [16]. SEM analysis revealed the TC-7 model to be a better mimic of *in vivo*-differentiated enterocytes as EPEC's ability to efface microvilli required EspF but not Map (Figure 3A–D) – as seen with the *ex vivo* model [16]. Thus, while the *espF* mutant could only ‘sink’ into the brush border (Figure 3C), wild type EPEC and *map* mutant strains induced a dramatic loss of peripheral microvilli in the TC-7 model (Figure 3).

We speculated that the aberrant effacement activity of Map in the Caco-2 model may be caused by an inability to down-regulate the Cdc42-dependent signalling triggered by this effector. As Cdc42-dependent activity of Map has been shown to be down-regulated by Tir/Intimin [26], effacement activity in strains deficient in Tir and/or Intimin were assessed with TC-7 cells. An EPEC triple mutant (lacking Tir, EspF and Intimin) was highly effective at effacing microvilli (Figure 3E) whereas a quadruple mutant (lacking Map, EspF, Tir and Intimin) was completely defective (Figure 3F), suggesting that Tir and Intimin was indeed suppressing this Map-dependent activity. Thus, in TC-7 cells, and presumably within *in vivo* enterocytes, Map can efface microvilli but only when Tir/Intimin are removed. Interestingly, we found that all strains deficient in Tir or Intimin exhibited an augmented ‘hyper-effacing’ ability to efface microvilli – dependent on the Map effector (similar to the triple mutant in Figure 3E; data not shown), suggesting Tir/Intimin strongly suppress Map-induced effacement. As this down-regulation of Map is not evident in the Caco-2 model [16], this may explain the differences between the cell lines.

### Microvilli effacement by Map and EspF is dependent on eukaryotic-like motifs

**Figure 4:**
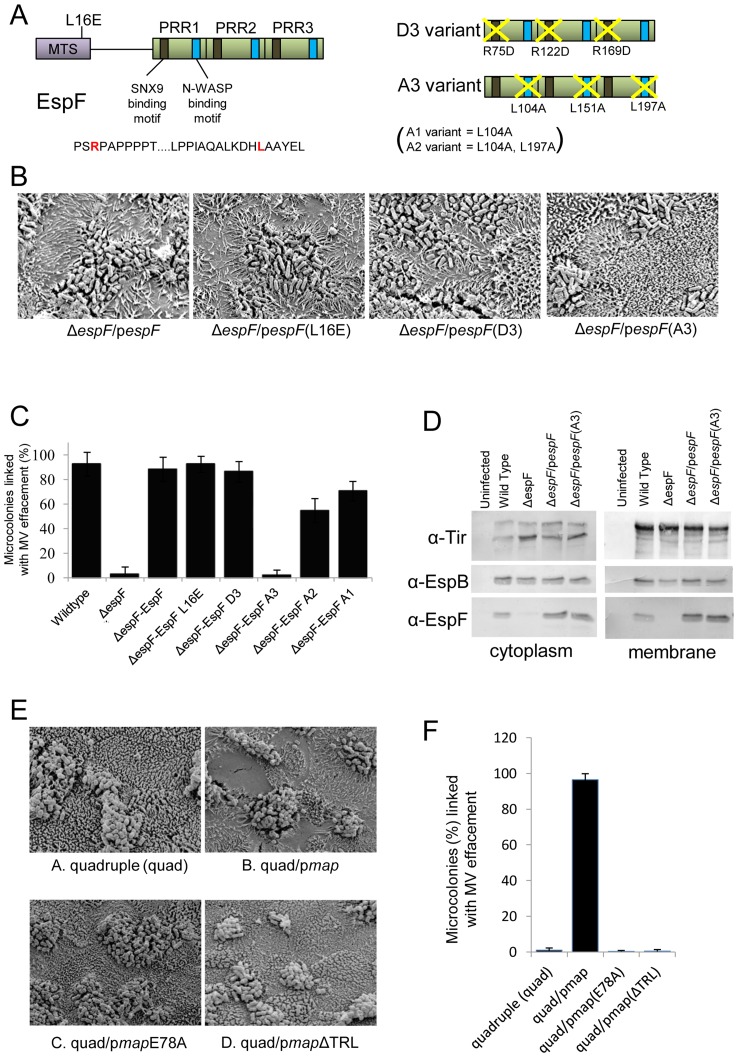
The EspF N-WASP binding motif is critical for effacement activity on TC-7 cells. Schematic of the EspF protein (A) showing proline rich repeat (PRR) C-terminal domain. Represntative sequence of the sorting nexin 9 (SNX9) and N-WASP binding motifs is shown with the critical arginine and leucine shown in red in red respectively (A). The diagram on the right shows the D3 EspF variant with the arginines of all 3 SNX9 motifs substituted for aspartic acid. The A3 variant has the critical leucines in all 3 N-WASP motifs replaced with alanine. Scanning electron microscopy revealed the *espF* gene could functional complement the *espF* mutant on TC-7 cell with no role for mitochondrial targeting or the SNX9 binding motif but a crucial role for multiple N-WASP binding motifs (B). Quantification of effacement was performed by assessing percentage of microvilli removed from the apical surface from 3 separate experiments, 5 fields of view per experiment (C). Data shows mean ± SE with p values given in the text. (D) Western blot analysis using antibodies against Tir, EspF and EspB in the cytoplasmic and membrane fractions of cells infected with the indicated EPEC mutants. (E) Scanning electron micrographs of TC-7 apical surface infected with the quadruple (quad; lacking Tir, Map, EspF and Intimin) complemented with the indicated plasmids. Effacement in (F) was quantified as in (C).

While both Map and EspF target mitochondria, with this function of EspF abolished by substituting leucine at position 16 to glutamic acid, their key subversive activities relate to extra-mitochondrial functions [13]. Thus, Map is a guanine exchange factor (GEF) that specifically activates Cdc42 in a manner dependent on a WxxxE motif while the final three residues, TRL, recruit the Ezrin binding protein 50 (Ebp50) to sustain Cdc42-dependent signalling [26], [27], [28], [29]. By contrast, EspF recruits and activates sorting nexin 9 (SNX9) and N-WASP to remodel host membranes [30], [31](Figure 4A). SNX9 binding motifs have been defined in each of three polyproline rich-repeat domains [30] with disruption of one (D1) having little impact on EspF's subversive activity while disrupting two (D2) or all three (D3) resulted in reduced or no subversive activity [30] (Figure 4A). The availability of variants lacking these EspF and Map features enabled complementation studies to define their involvement in the effacement process.

SEM analysis of infected TC-7 cells revealed that the effacement defect of the *espF* mutant was rescued by introducing plasmids expressing EspF or the L16E and D3 variants (Figure 4B and C). As putative N-WASP binding motifs have been defined for EspF homologues [32], substitutions were introduced to disrupt one (A1), two (A2) or all three (A3) recruitment motifs (see Materials and Methods; Figure 4A). While the A1 variant complemented the *espF* mutant defect (Figure 4C), the A2 variant displayed a significant defect and the A3 variant failed to provide detectable effacing activity (Figure 4B and C; p<0.0001). Indeed, the strain expressing the A3 variant had a similar defect as the Δ*espF* mutant (p = 0.99) suggesting the A3 variant was unable to complement the missing *espF* gene. Western blot confirmed that the two strains most defective for MV effacement (Δ*espF* and the A3 variant) were able to translocate effectors into host cells similar, if not better, than the wild type strain (Figure 4D). Thus, EspF's ability to efface microvilli in TC-7 cells depends on its N-WASP binding motif, with no apparent role for mitochondrial targeting or SNX9 recruitment.

While Map does not exhibit effacing activity in the TC7 or *ex vivo* models, its ability to hyper-efface microvilli in the absence of Tir/Intimin (see Figure 3) provided an opportunity to gain additional insight into its MV effacement activity. Map complementation studies with the EPEC quad mutant (lacking Map, EspF, Tir and Intimin) revealed a critical role for Map's WxxxE and -TRL motifs (Figure 4E and F), indicating that Map's effacing activity not only depends on its ability to activate Cdc42 but also to sustain Cdc42-dependent signalling [33]. Taken together this data suggests that effacement of the actin-rich microvilli by both Map and EspF proteins depends on eukaryotic-like motifs that are involved in actin nucleation and polymerisation events.

### The eukaryotic-like N-WASP binding motif of EspF is multi-functional

**Figure 5:**
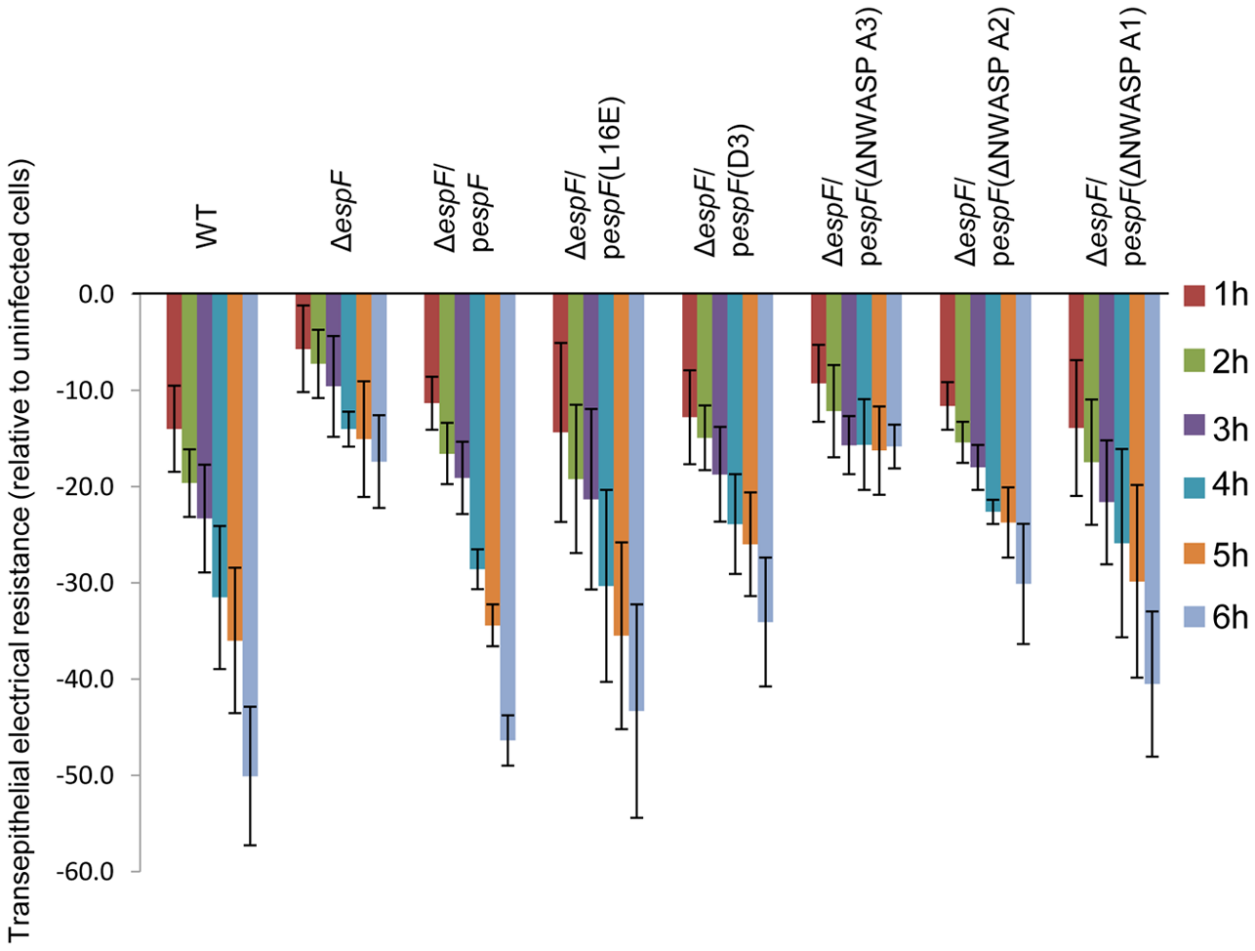
EspF-mediated epithelial barrier dysfunction of TC-7 cells requires the N-WASP binding motif. Loss of epithelial barrier function as measured by transepithelial electrical resistance (TER) of TC-7 cells infected for 6 h with the indicated EPEC strains. The various Δ*espF* mutants were complemented with Δ*espF* variants mutated in the mitochondrial targeting sequence (L16E), the SNX9 binding motif (D3), or 1, 2 or 3 of the N-WASP binding motif (A1, A2, A3 respectively). Data represents the mean ± SD for 3 independent experiments; p values given in the text.

As TC-7 cells provide a homogeneous model that mimic *in vivo* differentiated enterocytes, they were used to provide additional insight into another important *in vivo*-related event – epithelial barrier dysfunction. EspF is required for EPEC to disrupt the epithelial barrier [34] but the molecular mechanism is unknown. Complementation studies with the EspF variants revealed a critical role for EspF's N-WASP binding motifs (Figure 5). The Δ*espF* mutant defect was rescued by plasmid expression of native EspF or the L16E, D3 and A1 variants (p>0.05). By contrast, a partial defect was evident with the A2 variant while the A3 variant failed to induce barrier disrupting activity when complementing the Δ*espF* strain and thus was similar to the Δ*espF* mutant (p = 0.966). Thus, N-WASP recruitment by EspF appears to be multi-functional – inducing effacement of microvilli and causing a breakdown of the epithelial barrier in TC-7 cells.

## Discussion

Key advances in our understanding of host-pathogen interactions have come from the use of host-derived cell lines. For enteric pathogens, such as enteropathogenic *E. coli*, Caco-2 cells offer an attractive small intestinal model because of its morphological and physiological closeness to the *in vivo* state. Caco-2 cells are the most widely used small intestinal model [1], [2], employed in several diverse fields, yet their heterogeneity confers significant problems. In particular, Caco-2 cells that are sourced from different labs are likely to consist of vastly different cell populations as extrinsic factors such as passage time, seeding density and culture medium can favour certain subpopulations making results less reproducible. To alleviate these problems, Caco-2 clones have been isolated by various labs, combining the favourable physiological and morphological features of Caco-2 with the hope of greater experimental tractability and homogeneity.

Our previous work on the mechanism of microvilli effacement by enteropathogenic *E. coli* [16] revealed a discrepancy between Caco-2 cells and *in vivo* differentiated enterocytes. Thus, while the EPEC EspF effector was critical in inducing peripheral microvilli effacement in the *ex vivo* model, there were redundant roles for Map and EspF in Caco-2 cells. To gain further insight into the mechanisms of effacement, and obtain a tractable small intestinal model, we isolated and characterised 12 Caco-2 clones by limited dilution. Variation between the clones in growth rate, electrical resistance and transfection efficiency suggested our parent cell line was highly heterogeneous – a likely consequence of being derived directly from ATCC with few passages. Several clones shared growth, TER, transfection and surface morphologies with the TC-7 clone suggesting they may be derived from the same clonal cell type. However, as the TC-7 clone is well characterised [10] with a microvillar surface similar to *in vivo*-polarised cells and displayed the highest level of transfection, it was first to be examined in the MV effacement assay.

EPEC is known to cause mitochondrial dysfunction and swelling in HeLa cells [18]. In the present study, live cell imaging of infected TC-7 cells revealed a mitochondrial fusion event not seen in other cell types, that resulted in toroidal-shaped mitochondrial structures – dependent on EPEC expressing a functional effector delivery system. Toroidal mitochondria are reported to occur as a consequence of mitochondrial fusion between 2–3 mitochondria during cell stress and has been linked to their detachment from the cytoskeleton [35]. An increase in fusion or inhibition in fission of mitochondria can also lead to similar donut-shaped mitochondria [36], dependent on host proteins such as human PINK1 [36]. Although we found that such donut-shaped structures increased progressively during infection with EPEC, it is unclear whether they are specifically induced by the bacterium or whether they are a downstream consequence of effector activities. Many EPEC effectors disrupt the cytoskeleton which may inadvertently result in mitochondrial detachment leading to the increase in fusion as previously documented [36]. As toroidal mitochondria in EPEC-infected cells have not been visualised previously [18] [25], this highlights the benefits of real-time imaging with a polarised enterocyte monolayer. Live-cell imaging of TC-7 cells using other host specific markers may uncover more novel alterations during infection.

One of the major findings of this study was using TC-7 cells to uncover molecular events that underpin EPEC-induced microvilli effacement – a key feature of disease by this pathogen. Infected TC-7 cells behaved like *ex vivo*-derived intestinal tissue with an essential role for EspF but not Map in peripheral MV effacement. The absolute requirement of the EspF N-WASP binding motifs (NWBM) for microvilli effacement suggested that either directly or indirectly, N-WASP may be involved in maintaining the microvilli stability in the host cell. No role in the effacement process was found for EspF's sorting nexin 9 binding motif or mitochondrial targeting which have both been ascribed other specific functions during infection [31]. The essential role of the NWBM in effacement was strikingly similar to epithelial barrier dysfunction suggesting a related signalling pathway is subverted in both cases. Thus, EPEC employs the same function of EspF to trigger spatially and temporally distinct subversive events.

The regulation of effectors inside the host cell appears to be a tightly controlled event. We have previously shown that the EPEC proteins Tir and or Intimin regulate the functions of other EPEC effectors [26], [37]. In the present study, Map played no observable role in effacement in TC-7 cells, but removal of Tir and Intimin resulted in a Map-induced hyper-effacement that was entirely dependent on Map's C-terminal PDZ binding (-TRL) and WxxxE motif. Both these motifs are essential for Map's ability to activate Cdc42 [33], suggesting this pathway is involved in the hyper-effacement process. Thus, the molecular details that are responsible for effacement of microvilli are starting to be uncovered although paradoxically for Map and EspF, these appear to involve actin polymerisation and not depolymerisation functions. However, whether Cdc42 or N-WASP are involved directly or indirectly in preventing microvili destabilisation in the host cell is unclear.

We propose that TC-7 cells are a better model of *in vivo*-polarised enterocytes to investigate EPEC pathogenesis. They offer advantages including high transfection efficiency and densely packed brush border and crucially appear to mimic *in vivo*-related signalling events better than the parental Caco-2 cell line. The homogeneous nature of this clone would enable more accurate comparisons of data from different labs compared with the variable Caco-2 model and therefore will hopefully be adopted by others.
